# Reducing the residue of retractions in evidence synthesis: ways to minimise inappropriate citation and use of retracted data

**DOI:** 10.1136/bmjebm-2022-111921

**Published:** 2023-07-18

**Authors:** Caitlin Bakker, Stephanie Boughton, Clovis Mariano Faggion, Daniele Fanelli, Kathryn Kaiser, Jodi Schneider

**Affiliations:** 1 Dr. John Archer Library, University of Regina, Regina, Saskatchewan, Canada; 2 Research Integrity team, Editorial & Methods Department, Cochrane, London, UK; 3 Department of Periodontology and Operative Dentistry, Faculty of Dentistry, University Hospital Münster, Münster, Germany; 4 London School of Economics and Political Science, Dept. of Methodology, London, UK; 5 Heriot-Watt University, School of Social Sciences, Edinburgh Business School, Edinburgh, UK; 6 The University of Alabama at Birmingham, Birmingham, Alabama, USA; 7 School of Information Sciences, University of Illinois Urbana-Champaign, Champaign, Illinois, USA

**Keywords:** Retraction of Publication as Topic, Systematic Reviews as Topic, Evidence-Based Practice, Publishing, Information Storage and Retrieval

## Abstract

The incorporation of publications that have been retracted is a risk in reliable evidence synthesis. Retraction is an important mechanism for correcting the literature and protecting its integrity. Within the medical literature, the continued citation of retracted publications occurs for a variety of reasons. Recent evidence suggests that systematic reviews and meta-analyses often unwittingly cite retracted publications which, at least in some cases, may significantly impact quantitative effect estimates in meta-analyses. There is strong evidence that authors of systematic reviews and meta-analyses may be unaware of the retracted status of publications and treat them as if they are not retracted. These problems are difficult to address for several reasons: identifying retracted publications is important but logistically challenging; publications may be retracted while a review is in preparation or in press and problems with a publication may also be discovered after the evidence synthesis is published. We propose a set of concrete actions that stakeholders (eg, scientists, peer-reviewers, journal editors) might take in the near-term, and that research funders, citation management systems, and databases and search engines might take in the longer term to limit the impact of retracted primary studies on evidence syntheses.

**WHAT IS ALREADY KNOWN ON THIS TOPIC**

- Retracted publications continue to be incorporated into systematic reviews and other evidence syntheses without consideration of their retracted status.

**WHAT THIS STUDY ADDS**

- Authors, editors, publishers, research institutions, funding agencies and vendors can implement policies and practices to directly mitigate the inappropriate use of retracted publications in evidence synthesis.

**HOW THIS STUDY MIGHT AFFECT RESEARCH, PRACTICE OR POLICY**

- It suggests concrete actions regarding the conduct and evaluation of evidence syntheses, and the dissemination of any retractions.

## Introduction

The rate of evidence synthesis production has risen dramatically in recent years. Between 2000 and 2019, there was a 20-fold increase in the number of systematic reviews indexed, with a rate of 80 systematic reviews indexed per day by 2019.^1^ If not performed with rigour, this rapid proliferation of evidence syntheses poses varied challenges for the scientific community,^2^ and subsequently for downstream use in research and practice.

Retraction is an important mechanism for correcting the literature and protecting its reliability. Publications may be retracted for reasons related to unreliability of findings due to major error; fabrication or falsification or plagiarism or unethical research methods.^3^ While publications may sometimes be retracted for seemingly innocuous reasons, such as the journal publishing the wrong version or other administrative errors, studies suggest that between 22% and 54% of retractions are due to problems with methods or data, meaning that the reported results may not be valid.^4–8^ The remainder of retractions are due to other reasons, including plagiarism and other breaches of research integrity and ethics. While these may not directly affect the validity of reported results,^9^ they must be disincentivized nonetheless.

The number of retracted publications and the number of journals with a retraction policy have increased rapidly over the last two decades.^5 10^ This rise is unlikely to be caused by any increase in the prevalence of scientific misconduct, but instead may be due to the increasing ability and propensity of journals to retract problematic publications.^11^ This change may be the result of guidance issued by the International Committee of Medical Journal Editors (ICMJE) and the Council on Publication Ethics (COPE). ICMJE introduced guidance on handling retractions in 2004,^12^ which was subsequently expanded in 2013 and 2021.^13 14^ COPE has provided guidance on when a retraction may be necessary, how it should be undertaken and what should be included in the retraction notice.^3^ Although this guidance is available, it is not universally adopted. One study found that 10% of retraction notices contained inadequate information about the retraction.^15^ Another study, which alerted systematic review authors citing retracted clinical trials, found that 89% of those reviews were not corrected 1 year after notification.^16^

## The problem

Within the medical literature, the citation of publications continues after retraction. In one large-scale analysis of all open-access publications in PubMed, almost 50 000 citations to retracted publications were found, including 13 000 postretraction citations.^17^ Librarians and information specialists are well equipped to develop search strategies, which identify retracted research,^18–21^ and librarian involvement is associated with more reproducible systematic reviews.^22 23^ However, these information professionals are often underutilised or their efforts may not be appropriately described.^24–26^

The citation of retracted publications is not always problematic, as authors may be making note of publications, they have chosen not to include or may be discussing issues around retraction. However, evidence indicates that, in many cases, authors are not aware of the retracted status. Previous research has found that over 94% of postretraction citations in biomedicine do not mention or cite the retraction.^17^ Although the proportion of retracted publications in the biomedical literature may be small, their unknowing use in systematic reviews could have implications for patient care.

Recent evidence suggests that evidence syntheses mis-cite retracted studies at problematic levels and may not be immune to the impacts of some retractions. One study focusing on the citation of retracted publications in pharmacy systematic reviews found that 20% of the retracted publications had been cited in systematic reviews, and that approximately one-third of those citations had occurred after the retraction.^27^ Of these postretraction citations, 80% did not indicate that the publication had been retracted. These findings parallel other research, including a study of 587 systematic reviews and clinical practice guidelines that cite retracted randomised controlled trials (RCTs). A total of 135 of the citations occurred after the RCT was retracted, only 6% of which indicated that the RCT had been retracted.^28^ Another recent study of 229 meta-analyses found that 22% had included data from the retracted publication in their pooled summaries.^29^

According to the post hoc analysis discussed above,^29^ removing data associated with a retracted publication from the pooled summary did not alter results of most meta-analyses substantially. This suggests that results of systematic reviews are robust to individual retractions. However, the impact may be significant in some cases, especially when retractions are due to problems with methods, data or results. An expression of concern was published regarding one meta-analysis investigating the use of ivermectin in the prevention and treatment of COVID-19 after flaws in data collection and analysis were identified in two of the included studies.^30^ Exclusion of those two studies from the meta-analysis invalidated the review’s finding of decreased mortality. Additionally, a recent case study of a single clinical trial affected by data fabrication found that removing it would alter results of over half of the analyses in 22 meta-analyses on Apixaban. Reanalysis of the data, excluding data extracted from retracted publications, found that 87% of the affected estimates no longer favoured the intervention, while an additional 5% favoured the control.^31^

Whether or not retractions have an impact on the results of evidence synthesis, it is vital that the retracted status of a publication is acknowledged whenever the publication is cited. However, as shown above, evidence suggests that authors of systematic reviews and meta-analyses may be unaware of the retracted status of publications.

## Why is a publication’s retracted status often not acknowledged in evidence synthesis?

Although a journal may retract a publication, the communication of that retraction is often incomplete, both in the use of vague retraction notices with euphemistic language,^32–34^ and in the inconsistent and ineffective annotation of retracted publications.^35 36^

While the Retraction Watch database (retractiondatabase.org) includes over 36 000 retractions, and PubMed indexes over 13 000 records as ‘Retracted Publication’, it is highly likely that these are incomplete accounts.^37^ Studies have found that the retracted status of publications can vary significantly across different bibliographic databases.^38 39^ In addition to the limitations of databases, publications may not convey adequate signals of their retraction. For example, one study of retracted dental publications found watermarking to be inconsistently applied^40^; another found that only 39% of the retracted publications in emergency medicine had watermarking.^8^ Consequently, individuals arriving at the publication’s web page or downloadable file (such as a Portable Document Format (PDF) or Electronic Publication (EPUB) file) may not be aware of its retracted status.

While advancements have been made, such as the National Library of Medicine’s guidance to PubMed data providers^41^ and the incorporation of Retraction Watch data into citation managers such as Zotero, EndNote and Papers,^42 43^ these efforts are often led by a single platform or publisher. Although scholarly publishing is decentralised, widely adopted publication standards exist to facilitate discovery, data transfer and consistency across publications. A recently launched working group seeks to establish guidance for retractions^44^; however, currently no metadata and display standard are widely applied with regards to retracted publications, and the terminology and practices vary between publishers and bibliographic databases. The lack of consistency both within and across databases has been noted in previous research^45^ and while database providers should be acknowledged for their efforts to enhance discovery of the retracted status of publications, this lack of consistency is an impediment to meeting this objective.

The issues surrounding discovery of retracted publications do not account for the citation of publications that will later be retracted. The retraction process is lengthy, with an average time from publication to retraction of nearly 33 months.^46^ This has implications for their use. For example, retracted pharmacy publications cited in systematic reviews were retracted 7 years after publication, whereas retracted pharmacy publications that were not cited in systematic reviews were retracted 3.2 years after publication.^27^ Retractions may sometimes be preceded by an expression of concern, which aims to alert readers to a potential problem without definitively stating that the publication is flawed. However, expressions of concern remain controversial and are inconsistently used across scientific journals.^47^

## Proposals for mitigating the impact of retractions on evidence synthesis

How can the uptake of retracted publications in evidence synthesis be prevented? And how can the impact of retractions occurring after the publication of a review be reduced? Here we propose a set of concrete actions that various stakeholders might take in the near-term, and that research funders, database producers and search engine developers might take in the longer term.

### Authors of systematic reviews/meta-analyses should

- Ensure that the search strategy used will capture postpublication amendments, including retractions and expressions of concern, for example using methods described in the Cochrane Handbook^20 21^ and MECIR Manual.^19^
- Reassess publications of included studies just prior to publication of the systematic review, to capture retractions and expressions of concern issued while work on the review was underway. Functionality in citation managers, including current functionality in EndNote and Zotero, could streamline this process.^42 43^
- Acknowledge the retracted status of publications that are cited by explicitly noting that the publication has been retracted in the text and by indicating its retraction in the bibliography in accordance with guidance from citation style guides.^48–50^
- Authors who choose to cite retracted publications should offer a clear rationale for this decision. Whenever possible, clearly state the reason for retraction.
- Evaluate the impact that any retracted publication reporting results of an included study might have on the review’s conclusions regardless of the reason for retraction. Indeed, try to evaluate how influential any single study is to the overall results through sensitivity analyses.^51^
- Include a qualified information specialist or librarian with systematic review expertise on the research team. This is associated with several quality indicators for systematic reviews.^25 52^
- Always include ‘raw data’ alongside the publication (either statistical summaries of primary studies, or primary data, whichever were used) to allow independent robustness checks should there be a retraction after the review is published.
- Following the retraction of a cited publication, issue a statement either correcting the analyses or stating that no correction is necessary following reanalysis.

### Editors and publishers of journals publishing systematic reviews should

- Require authors to assess the methodology and robustness of individual studies included in the synthesis and require peer reviewers to check whether this is done.
- Require peer reviewers to assess the search strategy using methodological resources such as the Preferred Reporting Items for Systematic reviews and Meta-Analyses literature search extension checklist^53^ and the PRESS Guidelines^54^ and include information specialists and librarians as peer reviewers to help ensure that the retracted status of all publications of included studies is known.
- Incorporate a final stage into the workflow, for example, in the proofing stage, which involves a check of references just prior to publication. Check that no publications have been retracted or have had an expression of concern issued while the systematic review was under review or while it was in press. Consider making this system automated, potentially building on automated reference checking functionality in manuscript submission systems.^55–57^

### Editors of a journal retracting a publication should

- Alert publishers, journals and editors of any citing reviews by, for example, using backend data, particularly article identifiers, such as DOIs; citation databases, such as Web of Science and Scopus; and freely available data sources, such as COCI (the OpenCitations Index of Crossref open DOI-to-DOI citations) or OpenAlex.^58 59^
- Update metadata in applicable indexing systems, leveraging previously developed technical guidance.^41^
- Ensure that metadata schema and practices are in alignment with^60^Metadata 2020 principles,^60^ thereby enabling greater interoperability and reuse of higher quality metadata.

### Existing citation management software may be enhanced to

- Clearly display retraction status of publications, as seen in EndNote, Zotero and ReadCube Papers.^42 43 61^
- Format citations, when creating bibliographies, to indicate the retracted status of publications.
- Ensure that the retracted status of publications is transmittable to systematic review screening tools, such as Covidence and Rayyan.

### Existing databases and search engines may be enhanced to

- Clearly display retraction status of publications, with both human and machine-readable indicators, as already implemented in MEDLINE and Embase.
- Send automated alerts, on retraction of a publication, to those authors who have already cited it. These alerts could leverage the same technology as those described for journal editors retracting a publication who are seeking to set up alerts.
- Automatically deposit updated metadata in Crossref.
- Distinguish between publications of studies included in syntheses, those cited in other parts of the review and those cited as excluded due to the retraction. An example of this distinction is in effect in the Epistemonikos database.^62^

### Research institutions, funders and other supporting entities may

- Continue to raise awareness, share information and best practices by developing and delivering training and information resources to researchers and increasing awareness of existing resources, including previously developed training for information professionals.^63–67^
- Provide guidance on best practices for systematic reviews and meta-analyses and increase awareness of existing relevant guidance and initiatives.^19–21^
- Reinforce mechanisms to correct/update systematic reviews.^68 69^

All actors should continue to work to improve the incentives, norms, transparency, accuracy and efficiency of the mechanisms and processes of scientific self-correction and literature amendment.

## Conclusions

Many ‘unknown unknowns’ remain concerning the potential impacts of retracted publications on evidence synthesis and the best approaches to mitigate such impact. By reviewing the literature and offering a list of recommendations, our main objective is to stimulate further dialogue across all stakeholder groups and to inspire further concrete steps towards increasing awareness, promoting the continued implementation of existing guidance, creating standards and best practices and developing technical solutions to facilitate the expeditious handling of retracted publications. We hope that, over time, such practices will become a normal part of how science corrects itself, preserving confidence and integrity in the generation of knowledge.

## Data Availability

Data sharing not applicable as no datasets generated and/or analysed for this study.
